# Comparison of Various Generations of Superporous Hydrogels Based on Chitosan-Acrylamide and *In Vitro* Drug Release

**DOI:** 10.1155/2013/624841

**Published:** 2013-07-29

**Authors:** Shikha Bhalla, Manju Nagpal

**Affiliations:** ^1^Ganpati Institute of Pharmacy, Bilaspur, Yamunanagar, Haryana 135102, India; ^2^Chitkara College of Pharmacy, Chitkara University, Chandigarh-Patiala National Highway, Rajpua, Patiala, Punjab 140401, India

## Abstract

The aim of the current research work was to prepare and evaluate different generations of superporous hydrogels (SPH) of acrylamide and chitosan using gas blowing technique and evaluate them for swelling, mechanical properties, FTIR, SEM, XRD, and *in vitro* drug release. The ingredients used were acrylamide, N,N′-methylene bisacrylamide, chitosan, Pluronic F127, ammonium per sulfate-N,N,N′,N′-tetramethylenediamine, and sodium bicarbonate. All ingredients were mixed sequentially with thorough stirring. The effect of different drying conditions on properties of SPH was also evaluated. Ethanol treated batched showed maximum swelling properties due to uniform pores as indicated in SEM studies. Equilibrium swelling time was less than 10 min in all batches. Freeze drying led to lowering of density which is also supported by porosity and void fraction data. Maximum mechanical strength was found in superporous hydrogel interpenetrating networks due to crosslinked polymeric network. 70% drug was released at the end of 2 h, and further the release was sustained till the end of 24 h. *In vitro* drug release kinetics showed that drug release occurs by diffusion and follows Super Case II transport indicating that mechanism of drug release is not clear. Superporous hydrogel interpenetrating networks can be successfully used as sustained release gastroretentive devices.

## 1. Introduction

With the advent of new generation drugs, increasing attention is being given to the new methodologies for sustained and better control of drug administration (as discussed by Risbud and Bhonde [1]). Site specific delivery using novel formulation designs would improve local therapy in GI tract, optimize systemic absorption, and minimize premature drug degradation. However, the inability to confine the dosage form in desired area of GIT is one of major difficulties (as discussed by Patel and Amiji [2]). Various technical advancements in fabrication of dosage forms have been explored in this area to retain the dosage form in upper part of gastrointestinal (GI) tract. Small transit time of the dosage form is the main factor responsible for suboptimal absorption making traditional extended release development challenging. A variety of systems such as floating systems, bioadhesive systems, raft systems, expanding systems, swelling systems, and low-density systems have been fabricated as gastroretentive drug delivery (as discussed by Rocca et al. [3] and Bardonnet et al. [4]). Hydrogel polymers are structurally loosely cross-linked networks which have the ability to absorb considerable amounts of water or aqueous fluids to form a stable hydrogel, and the absorbed water is hardly removed under some pressure (as discussed by Peppas et al. [5]). Advancement in hydrogels, that is, superporous hydrogels (SPH), has been explored using acrylamide based matrices in 1999 for pharmaceutical drug delivery applications. Superporous hydrogels (average pore size >100 *μ*m) swell to equilibrium size within a minute due to rapid water uptake by capillary channels through numerous interconnected open pores (as discussed by Chen et al. [6]). Further superporous hydrogel composites (SPHCs) (as discussed by Park et al. [7–10]) or by the formation of interpenetrating (as discussed by Qiu and Park [11]) or hybrid hydrogel network (as discussed by Omidian et al. [12]) have been developed with enhanced mechanical strength. Interpenetrating polymer networks (IPN) are a mixture of two or more cross-linked networks that are dispersed or mixed at a molecular segmental level. Interpenetrating polymer network (IPN) could help improve the mechanical strength and resiliency of the polymer (as discussed by Omidian et al. [13, 14]). Chitosan, a deacetylated product of chitin, also has been used in controlled delivery systems using many drugs (as discussed by Hejazi and Amiji [15] and Bonina et al. [16]). Ranitidine is a competitive reversible inhibitor acting on histamine H_2_ receptors, including receptors on gastric cells with a minimal effect on H_1_ receptors. It is mainly used for the treatment of active duodenal ulcers, gastric ulcers, Zollinger-Ellison syndrome, gastroesophageal reflux disease, and erosive esophagitis (as discussed by Sharma et al. [17]). The indicated oral dosage of ranitidine is 150 mg twice daily or 300 mg once daily. The drug has a short biological half-life of approximately 2-3 h and is absorbed only in the initial part of the small intestine. Colonic metabolism of ranitidine is also responsible for poor bioavailability of the drug. The present study explored the feasibility of superporous hydrogel interpenetrating networks based on polyacrylamide-chitosan for controlled delivery of ranitidine hydrochloride. 

## 2. Material and Methods

### 2.1. Materials

Ranitidine was obtained as a gift sample from Alkem Labs, Baddi, India. Chitosan was kindly gifted by the Central Fisheries Institute, Cochin. Acrylamide (AM), Acrylic Acid (AA), and Sodium Bicarbonate (NaHCO_3_) of analytical grade were used (Loba Chemie, Mumbai), and Pluronic (PF127) was purchased from Sigma Aldrich, Steinheim. N,N′-Methylene bisacrylamide (BIS) was procured from Central Drug House Pvt. Ltd., New Delhi. Glutaraldehyde, ammonium persulphate (APS), and N,N,N′,N′-tetramethylethylenediamine (TEMED) (Loba Chemie, Mumbai) were also used in the study.

### 2.2. Synthesis of SPH (Gas Blowing Technique)

Superporous hydrogels were synthesized using gas blowing technique (as discussed by Chen et al. [6]). Acrylamide (AM) was used as a monomer in the preparation of conventional superporous hydrogels. All the ingredients acrylamide, N,N′-methylene bisacrylamide (BIS), water, Pluronic F127 (foam stabilizer), and acrylic acid were added sequentially in a test tube (15 mm diameter) followed by the addition of ammonium persulfate (APS). The solvent used for the preparation of monomer solution was deionised, distilled water. The test tube was thoroughly shaken after the addition of each ingredient. This was followed by the addition of N,N,N′,N′-tetramethylene diamine solution (TEMED) with constant shaking. Finally, 100 mg of sodium bicarbonate was added into the test tube with vigorous shaking which led to foaming and gelation. The reaction leads to formation of conventional superporous hydrogels (first generation superporous hydrogels, CSPH). 

#### 2.2.1. Effect of Pretreatment (Addition of Chitosan/Crosslinked Chitosan)

Superporous hydrogel composites (second generation superporous hydrogels, SPHC) were prepared by addition of second polymer, that is, chitosan, in the primary polymer mixture before the addition of APS-TEMED (as discussed by Bonina et al. [16]). Chitosan was added in two different ratios (AM : CS) of 1 : 1 and 1 : 1.5 to evaluate the effect on swelling and mechanical strength of SPHC.

Superporous hydrogel interpenetrating networks (third generation superporous hydrogels, SPHIPN) were prepared by the addition of chitosan and glutaraldehyde solution in reaction mixture (*in situ* crosslinking of chitosan occurs) (as discussed by Bonina et al. [16]). Chitosan was added in two different ratios (AM : CS) of 1 : 1 and 1 : 1.5 to evaluate the effect on swelling and mechanical strength of SPHIPN while keeping glutaraldehyde concentration constant.

The superporous hydrogels were retrieved from the test tubes. The composition of various generations of SPH is given in Table 1. 

#### 2.2.2. Effect of Posttreatment and Drying Conditions

Superporous hydrogels of all generations (CSPH, SPHC, and SPHIPN) were given different posttreatments and drying conditions:

- ethanol dehydration followed by oven drying at 55°C,
- simulated Gastric Fluid (SGF) treated followed by oven drying at 55°C,
- freeze drying.

Various SPHs formulations with different pre- and posttreatments are shown in Table 2. 

### 2.3. Physical Evaluation of Various SPH

Various samples (without drug) were evaluated for swelling, porosity, compression, and SEM studies to get the promising batches for further studies.

#### 2.3.1. Equilibrium Swelling Ratio and Swelling Time

Completely dried superporous hydrogel samples were weighed and kept in excess of swelling medium (distilled water at 37°C) until the equilibrium swelling (as discussed by Chen and Park [9]) was achieved and the hydrogel samples were again weighed. The time required for equilibrium swelling was noted as the equilibrium swelling time.

#### 2.3.2. Density Measurement

Solvent displacement method (as discussed by Chavda and Patel [18, 19]) was used for the determination of density. The preweighed superporous hydrogel samples were immersed in hexane in a graduated cylinder. Initial volume of hexane was noted and the increase in volume was also noted. Density was calculated as follows:

(1) $$\begin{array}{c} \text{Density}=\frac{\text{Mass}}{\text{Volume}\;\;\text{of}\;\;\text{solvent}\;\;\text{displaced}}. \end{array}$$

#### 2.3.3. Determination of Void Fraction

It is determined by immersing the superporous hydrogel samples in HCl (pH 1.2) up to equilibrium swelling (as discussed by Qiu and Park [11]). Dimensions of swollen superporous hydrogel samples were measured as dimensional volume. Void fraction was calculated as follows:

(2) $$\begin{array}{c} \text{Void}\;\;\text{fraction}=\frac{\text{Dimensional}\;\;\text{volume}\;\;\text{of}\;\;\text{hydrogel}}{\text{Total}\;\;\text{volume}\;\;\text{of}\;\;\text{pores}}. \end{array}$$

Total volume of the pores was calculated by subtracting the weight of the dried hydrogel from the weight of the swollen hydrogel.

#### 2.3.4. Porosity Measurement

Superporous hydrogel samples were immersed in hexane overnight and weighed when excess amount of hexane on surface was removed (as discussed by Qiu and Park [11] and Vishal Gupta and Shivakumar [20]). Porosity was calculated as follows:

(3) $$\begin{array}{c} \text{Porosity}=\frac{V_{p}}{V_{t}}. \end{array}$$

*V*_*t*_ is the total volume of SPH, *V*_*p*_ is the pore volume of SPH (*V*_*t*_ − *V*_SPH_), and *V*_SPH_ is the volume of liquid displaced.

#### 2.3.5. Mechanical Properties

The compression force (N) was determined using TA.TXPlus Texture Analyzer (Stable Micro Systems) (as discussed by Sharma et al. [17]) using a cylindrical aluminum probe (P_75_) having a pretest speed of 2.00 mm/sec, test speed of 1 mm/sec, and posttest speed of 2 mm/sec up to a distance of 3 mm. The swollen superporous hydrogel samples were placed on a disk shaped platform. Compression force was estimated as the peak value in the force-time plot.

#### 2.3.6. Scanning Electron Microscopy (SEM)

The dried superporous hydrogel samples were cut in transverse section and mounted on a double sided tape on aluminium stubs and were sputter coated with gold using fine coat ion sputter (JEOL) and then micrographs were recorded using scanning electron microscope (JEOL JSM-6100) to study the effect of treatment and drying conditions on the porous nature of superporous hydrogels.

### 2.4. Drug Loading into SPHIPN

Various SPHIPN were loaded with drug (ranitidine hydrochloride, 150 mg) using the method of soaking or equilibration. The amount of water required for complete swelling of SPHIPN was determined, and thereafter drug was dissolved in the predetermined amount of water (as discussed by Chavda and Patel [18]). The dried samples were kept in the drug solution and left until all the solution was absorbed. Finally, the completely swollen hydrogel samples were freeze dried for 24 h. Six formulations (A1–A6) were prepared in which A1–A3 batches with AM : CS ratio of 1 : 1, and drug to HPMC ratio varies from 1 : 0, 1 : 0.5, 1 : 1, respectively, and A4–A6 with AM : CS ratio of 1 : 1.5 and drug to HPMC varies from 1 : 0, 1 : 0.5 and 1 : 1, respectively. Composition of various drug loaded batches is given in Table 3.

### 2.5. Evaluation of Drug Loaded SPHIPN

#### 2.5.1. Fourier Transform Infrared Spectroscopy (FTIR)

Freeze dried drug loaded hydrogel sample was powdered in a mortar. FTIR spectra of pure drug, acrylamide, HPMC, chitosan, and drug loaded SPHIPN were recorded using KBr pellet over the range of 400–4000 cm^−1^ using (Perkin Elmer Spectrum 400) FTIR spectrophotometer. The FTIR spectrum was recorded to check the interaction of drug with polymeric hydrogel network.

#### 2.5.2. X-Ray Diffraction Analysis (X-RD)

The X-ray powder diffraction was done to monitor the changes in crystallinity characteristics of the drug when the drug was loaded into superporous hydrogel interpenetrating networks. The freeze dried drug loaded hydrogel sample was powdered in mortar and then the XRD patterns were measured using X-ray diffractometer (X'pert PRO, PAN analytical, The Netherlands) using Ni filtered, CuK *α* radiation with a voltage of 45 kV and 40 mA current.

#### 2.5.3. Drug Loading Capacity

Drug loading capacity of SPHIPN was determined by dissolving preweighed dried triturated superporous hybrid hydrogel sample in 50 mL of simulated gastric fluid (SGF) (pH 1.2), and the solution was filtered and analyzed using UV-VIS spectrophotometer at 220 nm. 

#### 2.5.4. *In Vitro* Drug Release Studies

The *in vitro* drug release of selected batches of SPHIPN was estimated in triplicate using the United States Pharmacopoeia (USP) Dissolution Type II apparatus (Paddle Type) at 37 ± 0.5°C at a paddle speed of 50 rpm in 900 mL of SGF (pH 1.2) for 24 h. At regular intervals 10 mL of the dissolution, medium was withdrawn and an equivalent volume of fresh dissolution medium was replaced. The samples were analyzed for the drug concentration using UV-VIS spectrophotometer (Shimadzu, Japan) at 220 nm. 

## 3. Results and Discussions

### 3.1. Equilibrium Swelling Ratio and Swelling Time

**Figure 1:**
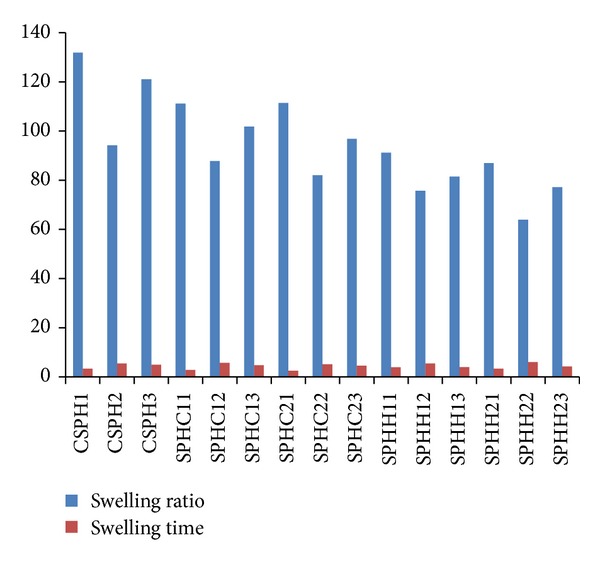
Equilibrium swelling ratio and equilibrium swelling time of various batches of superporous hydrogels.

Figure 1 shows the results of equilibrium swelling ratio and equilibrium swelling time.

(i) *Effect of Addition of Chitosan (SPHC) and Crosslinked Chitosan (SPHIPN).* Maximum swelling ratio was observed in CSPH1. The physical mixing of chitosan in acrylamide network (SPHC11) led to a decrease in swelling ratio which was further decreased by *in situ* crosslinked chitosan (SPHIPN11). The increase in chitosan ratio (SPHC21 and SPHIPN21) however did not significantly affect the swelling ratio. 

The equilibrium swelling time was decreased by physical addition of chitosan (3.4 min. in CSPH1, 2.8 min. in SPHC11), whereas SPHIPN11 showed again 3.8 min. for equilibrium swelling. The physical mixing of chitosan supports capillary channels of polymer network. No effect on equilibrium swelling time was observed by the increase in chitosan ratio.

(ii) *Effect of Posttreatments and Drying Conditions.* Ethanol treated oven dried samples (CSPH, SPHC, and SPHIPN) showed higher swelling ratios than other treatment and drying conditions. Ethanol replaces all the water within polymeric network, and hydrogels become condensed and hardened. Further drying in oven did not collapse the pores because ethanol is less dense than water (present in very small or negligible fractions). Significant decrease in equilibrium swelling ratio was observed in SGF treated batches (CSPH2, SPHC12, and SPHIPN12) which may be due to the reason that the SGF treatment partially protonized the anionic SO_3_^−^ group into the SO_3_H group (acidification). Freeze drying (CSPH3, SPHC13, and SPHIPN13) also led to decrease in equilibrium swelling ratio which may be due to slight distortion in porous structure during the process of drying (as water is sublimed in this process). But it was not that much significant as in SGF treated batches. 

Maximum equilibrium swelling time was observed in SGF treated batches (CSPH2, SPHC12, and SPHIPN12) followed by freeze dried hydrogels and then ethanol treated hydrogels (CSPH1, SPHC11, and SPHIPN11). The least equilibrium swelling time in ethanol treated may be due to preserved capillary channels by ethanol treatment. Formulations with increased chitosan ratios (SPHC21, SPHC22, and SPHC23; SPHIPN21, SPHIPN22, and SPHIPN23) follow the same effect pattern on equilibrium swelling ratio and time.

### 3.2. Density, Void Fraction, and Porosity

**Figure 2:**
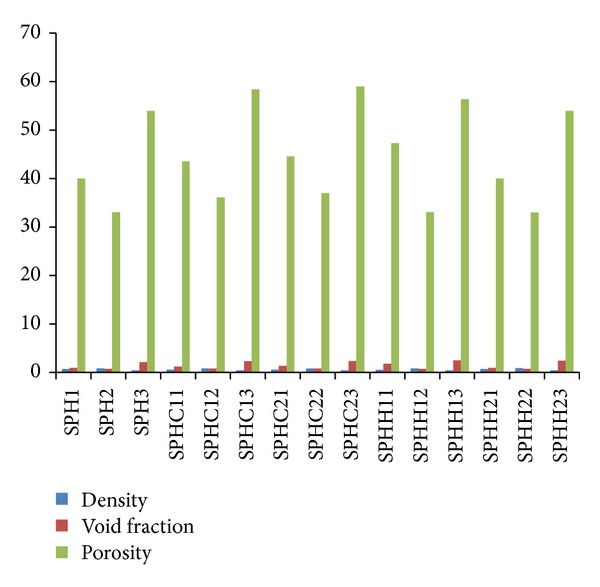
Density, porosity, and void fraction data of various batches of superporous hydrogels of different generations.

Figure 2 shows the results of density, void fraction, and porosity of various SPH.

(i) *Effect of Addition of Chitosan (SPHC) and Crosslinked Chitosan (SPHIPN).* All the hydrogels (CSPH, SPHC, and SPHIPN) exhibit density of less than 1. Therefore, these can act as floating devices for gastroretentive delivery. The addition of chitosan/crosslinked chitosan (SPHC11/SPHH11) slightly decreases the density values but no major change was observed. No change was observed in void fraction in values by addition of chitosan/crosslinked chitosan (SPHC11, SPHIPN11 as compared to CSPH1). Porosity values increased by addition of chitosan/crosslinked chitosan (higher in SPHC11 and SPHIPN11 than CSPH1) but were not observed as significant.

(ii)* Effect of Different Drying Conditions.* All the hydrogels exhibit density values of less than 1. Ethanol treated oven dried hydrogels (CSPH1, SPHC11, and SPHIPN11) exhibit low density values as compared to SGF treated oven dried hydrogels (CSPH2, SPHC12, and SPHIPN12). Freeze drying led to very lighter hydrogels (CSPH3, SPHC13, and SPHIPN13) as indicated by sharp decrease in density as compared to oven drying. Maximum void fraction values were observed in freeze dried hydrogels and least values were in case of SGF treated hydrogels. The same results were observed in porosity data of the hydrogels. The ethanol treatment leads to rigid SPHs (due to precipitation of polymer chains in poor solvent) which contributed to better maintenance of pore structures and less shrinkage of polymer network during drying in oven. The uniformity in pores with interconnected channels is also supported by SEM analysis. 

### 3.3. Mechanical Properties

**Figure 3:**
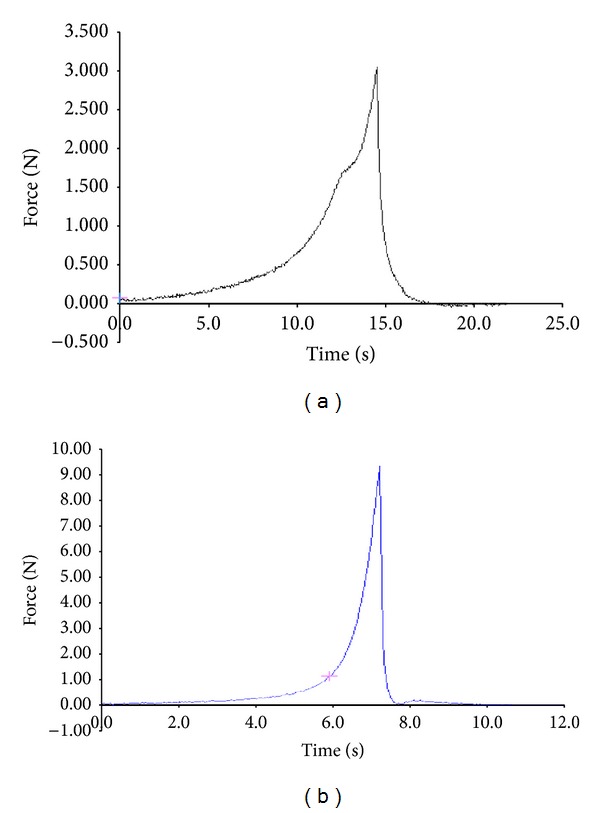
Force versus time plots of superporous hydrogels: (a) SPHC and (b) SPHIPN.

The mechanical strength studies were carried out on mainly ethanol treated hydrogel samples (CSPH1, SPHC11, and SPHIPN11). Addition of other polymer (chitosan) in primary polymeric network (acrylamide) was done to enhance the mechanical strength of hydrogels. The fracture of CSPH1 was observed at initial level during compression studies using texture analyzer. SPHC maintained their integrity at higher compression force between 3 and 4 N (Figure 3(a)) and tend to break or fragile after increasing the force; SPHIPN were found to be elastic enough that they did not break under 10 N of compression force and push the probe back with a force (Figure 3(b)). 

SGF treated hydrogels (CSPH2, SPHC12, and SPHIPN12) were not selected because of their compromising swelling characteristics. 

Freeze dried samples (CSPH3, SPHC13, and SPHIPN13) also possess equivalent elasticity and compression as with ethanol treated samples.

### 3.4. Scanning Electron Microscopy (SEM)

**Figure 4:**
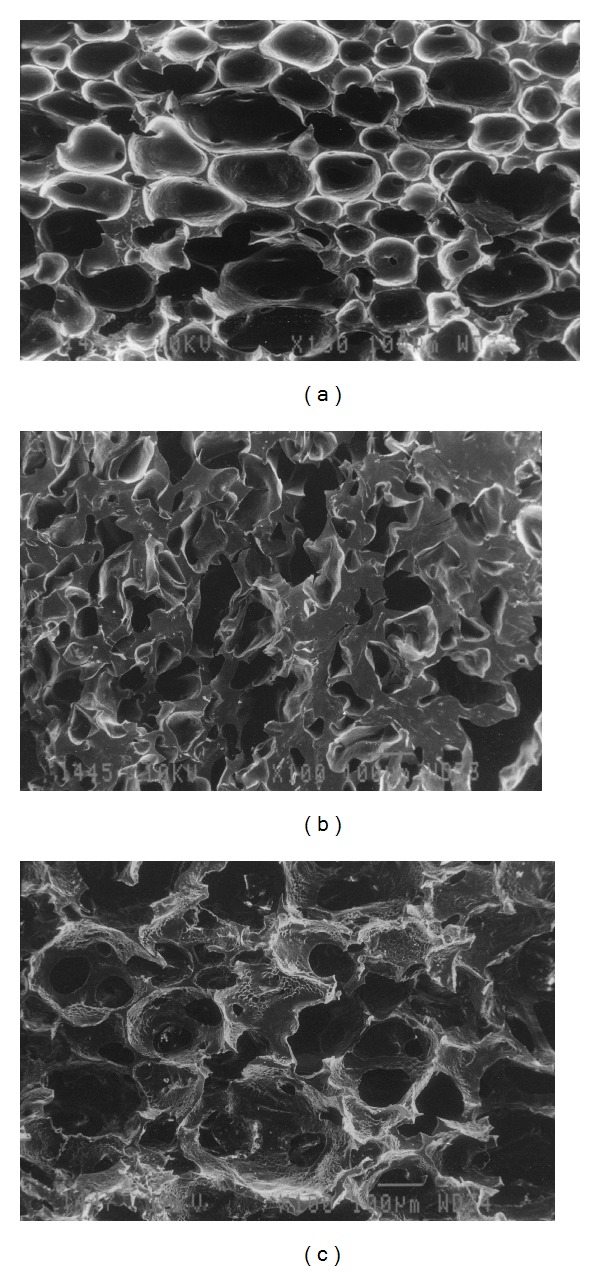
Scanning electron micrographs of superporous interpenetrating networks: (a) ethanol treated, (b) SGF treated and (c) freeze dried.

Addition of chitosan/crosslinked chitosan did not affect the porous network of polymeric hydrogel structure, whereas different posttreatments and drying conditions significantly affect the porous network. Only SPHIPN were selected for SEM analysis. The ethanol treated batches showed the uniform distribution of the pores in the hydrogel network and maintained capillary channels were also observed (Figure 4(a)). Treatment with SGF resulted in poor porous network which may be due to the tightly bonded chains in the structure due to the protonation of anionic SO_3_^−^ group at surface of hydrogel (Figure 4(b)). SEM images also revealed the presence of pores in freeze dried batches but presence of distorted capillary channels was also observed (Figure 4(c)). 

### 3.5. Fourier Transform Infrared Spectroscopy (FTIR)

**Figure 5:**
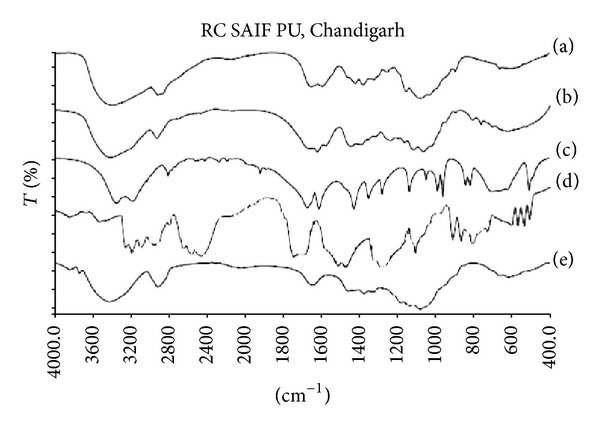
Overlay diagram of FTIR spectrum of (a) HPMC; (b) chitosan; (c) acrylamide; (d) ranitidine hydrochloride; (e) superporous hydrogel interpenetrating network.

The IR spectrum of ranitidine hydrochloride (Figure 5) showed the characteristic peak at 2510 cm^−1^ (attributed to the N^+^–H bond in protonated tertiary amine group), 1618.6 cm^−1^ (stretching vibration of C=N), 1470.4 cm^−1^ and 1262.02 cm^−1^ (stretching vibration of nitro group attached to saturated carbon), stretching vibration of amine at 3192.07 cm^−1^, and other characteristic peak of N=O was found at 1421.14 cm^−1^. The FTIR spectra of chitosan showed a characteristic band of 3370 cm^−1^ which is attributed to amino and hydroxyl groups stretching vibration; characteristic band of carbonyl was seen at 1653 cm^−1^. The FT-IR spectra of acrylamide showing the presence of band at 3354.19 cm^−1^ can be assigned to symmetrical and asymmetrical stretching of N–H group. The characteristic C=O stretching vibration bands of amide and acid groups have been observed at 1673.6 cm^−1^ and 1720 cm^−1^, respectively, and the peak for –CH=CH_2_ group at 1620 cm^−1^ was observed. The FTIR spectra of SPHIPN showed the presence of the characteristic peaks of the drug showing that there was no drug polymer interaction. Formation of a new peak in FTIR spectrum of drug loaded SPHH at 1610 cm^−1^ proved the crosslinking between glutaraldehyde and chitosan.

### 3.6. X-Ray Diffraction Analysis (X-RD)

**Figure 6:**
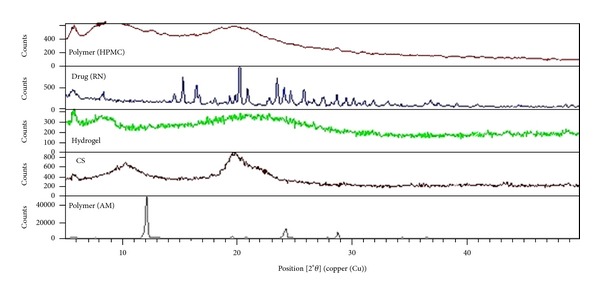
Overlay diagram of X-RD of HPMC; pure drug; superporous hydrogel interpenetrating network; CS: chitosan; AM: acrylamide.

XRD pattern of ranitidine (Figure 6) showed sharp crystalline peaks and the maximum at 20.3°, and other sharp peaks were found at 2*θ* = 23.5°, 15.3°, and 16.5°. The XRD pattern of chitosan showed broad diffraction peaks at 2*θ* = 10° and 21° which are typical finger prints of semicrystalline chitosan. XRD pattern of SPHIPN showed most of the characteristic peaks of drug. Absence of some peaks may be due to presence of excess polymers with the drug responsible for reduced crystallinity of drug.

### 3.7. Drug Loading Capacity

The drug loading capacity was found to be more than 97% in all SPHIPN indicating these systems act as good reservoir devices for drugs.

### 3.8. *In Vitro* Drug Release Studies

**Figure 7:**
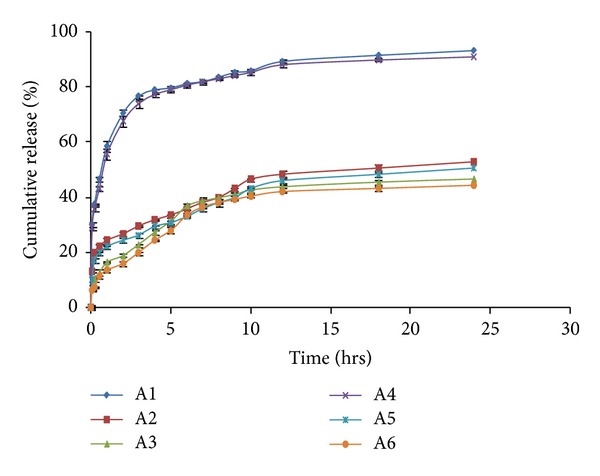
Comparative *in vitro* drug release of various formulation batches (A1–A6).

*In vitro* drug release was carried out in all the drug loaded batches (A1–A6). Initially, drug release was found to be about 70.67% and 67.78% (batch A1 and A4) at the end of 2 h (Figure 7). Further, the drug release was sustained up to 24 h (93.10% and 91.01% in batch A1 and A4, resp.). The initial burst release may be due to the presence of free drug on the surface of hydrogel and later on drug release occurs through diffusion; that is, the drug incorporated in the hydrogel network was released slowly. Addition of HPMC leads to further sustain the release of drug from the hydrogel (46.53% and 44.27% in batch A2 and A3 at the end of 24 h). It was also found that the increase in concentration of chitosan (batch A5 and A6) did not affect the release considerably.

### 3.9. Drug Release Kinetics

The drug release data was studied to understand the release mechanism. The values of regression coefficient for various kinetic models are shown in Table 4. *In vitro* drug release follows Higuchi model indicating drug release occurs by diffusion. The value of the release exponent in Korsmeyer model was more than 0.85 which is beyond the limits of Korsmeyer model so called power law or Super Case II transport indicating that mechanism of drug release is not clear.

## 4. Conclusion

Different generations of SPH have been evolved to address the need for pharmaceutical applications such as gastroretentive drug delivery. SPHs with enhanced mechanical properties were prepared by introducing another polymer network and increased the feasibility of using them as oral sustained release devices particularly for gastric retention. The interconnected pore structures are not destroyed by penetrating polymer networks thereby maintaining high and fast swelling characteristics with improved mechanical properties.

## Figures and Tables

**Table 1:** Composition of various formulation batches of superporous hydrogels of different generations.

Ingredients	CSPH	SPHC (AM : CS = 1 : 1)	SPHC (AM : CS = 1 : 1.5)	SPHH (AM : CS = 1 : 1)	SPHH (AM : CS = 1 : 1.5)
Acrylamide	1000	500	500	500	500
BIS (2.5%)	200	200	200	200	200
Water	460	—	—	—	—
PF127 (10%)	100	100	100	100	100
Acrylic acid	45	45	45	45	45
CS	—	500	750	500	750
Glutaraldehyde (50%)	—	—	—	10	10
APS (20%)	40	40	40	40	40
TEMED (20%)	40	40	40	40	40
NaHCO_3_ (mg)	90	90	90	90	90

*All the quantities (except NaHCO_3_) are expressed in *μ*L.

**Table 2:** Formulation codes of different batches of superporous hydrogels based on posttreatment and drying conditions.

Batch	Posttreatment	Drying conditions
CSPH1	Ethanol	Oven drying at 55°C
CSPH2	SGF	Oven drying at 55°C
CSPH3	—	Freeze drying

SPHC11	Ethanol	Oven drying at 55°C
SPHC12	SGF	Oven drying at 55°C
SPHC13	—	Freeze drying
SPHC21	Ethanol	Oven drying at 55°C
SPHC22	SGF	Oven drying at 55°C
SPHC23	—	Freeze drying

SPHIPN11	Ethanol	Oven drying at 55°C
SPHIPN12	SGF	Oven drying at 55°C
SPHIPN13	—	Freeze drying
SPHIPN21	Ethanol	Oven drying at 55°C
SPHIPN22	SGF	Oven drying at 55°C
SPHIPN23	—	Freeze drying

**Table 3:** Composition of various drug loaded batches of superporous hydrogel interpenetrating networks.

Formulation batch	Drug (mg)	Acrylamide (*µ*L)	Chitosan (*µ*L)	HPMC (mg)
A1	150	500	500	—
A2	150	500	500	75
A3	150	500	500	150

A4	150	500	750	—
A5	150	500	750	75
A6	150	500	750	150

**Table 4:** Regression coefficient values of various release kinetic models.

Formulation batch	Zero order	First order	Higuchi model	Korsmeyer-Peppas model	
	*R* ^2^	*R* ^2^	*R* ^2^	*n*	*R* ^2^
A1	0.48	0.817	0.8	3.744	0.979
A2	0.783	0.877	0.965	4.105	0.972
A3	0.699	0.755	0.913	2.668	0.976
A4	0.406	0.772	0.801	3.792	0.977
A5	0.751	0.874	0.963	3.611	0.972
A6	0.715	0.757	0.911	2.4	0.965
